# The rare thoracic complication: perforation of gastric fundus ulcer: a case report

**DOI:** 10.1186/s13256-022-03684-1

**Published:** 2022-12-22

**Authors:** I. S. Polyakov, A. L. Kovalenko, A. N. Petrovsky, A. V. Akobyan, V. A. Porhanov

**Affiliations:** 1 Oncological Department, Scientific Research Institute – Ochapovsky Regional Clinical Hospital no. 1, Krasnodar, Russia; 2 Department of Thoracic Surgery no.2, Scientific Research Institute – Ochapovsky Regional Clinical Hospital no. 1, Krasnodar, Russia; 3 Surgical Department, Scientific Research Institute – Ochapovsky Regional Clinical Hospital no. 1, Krasnodar, Russia; 4 Department of Thoracic Surgery, Scientific Research Institute – Ochapovsky Regional Clinical Hospital no. 1, Krasnodar, Russia

**Keywords:** Perforation of a gastric ulcer, Pleural empyema, Gastropleural fistula

## Abstract

**Background:**

Gastropleural fistula is an exceptionally rare condition, the incidence of which is currently unknown (Kunieda *et al.* in Intern Med 51(3):331, 2012, https://doi.org/10.2169/internalmedicine.51.6823, Iqbal *et al.* in Cureus 11(2):e4136, 2019, https://doi.org/10.7759/cureus.4136, Kathayanatt *et al.* in Lung India 37(2):174–175, 2020, https://doi.org/10.4103/lungindia.lungindia_242_17). The etiology varies from traumatic or iatrogenic injury to perforation in a herniated stomach due to ischemia, ulceration, or malignancy.

**Case presentation:**

A 27-year-old European male presented to our hospital with complaints of general weakness and shortness of breath. The patient had a single episode of hemoptysis before admission. A computed tomography scan demonstrated a left-sided pyopneumothorax, a defect in the left main bronchus, and signs of pneumonia in the lower sections of the right lung. Therefore, a rare complication of perforation of a gastric fundus ulcer with the formation of a subdiaphragmatic abscess, gastropleural fistula, gangrene of the left lung with circular necrosis of the left main bronchus and diastasis of its edges, and pleural empyema on the left is presented in this report.

**Conclusions:**

Although, a radical surgery may be preferable for this suspected malignancy; it should be weighed carefully against the risk of sepsis and the morbidity associated with a prolonged procedure in a sick patient. Damage-control surgery may be a viable option for a very sick patient, with more extensive resection reserved for later, provided the risk of infection and bleeding has been mitigated.

## Background

The prognosis of patients with a gastropleural fistula is poor. However, surgical management involving a laparotomy and potentially a thoracotomy has been described depending on the underlying etiology [1–3]. At present, a limited number of cases have been submitted to the medical databases.

## Case presentation

A 27-year-old European male presented to our hospital with complaints of general weakness and shortness of breath. He had a single episode of hemoptysis before admission. The initial blood investigations revealed a total leukocyte count of 44.61 × 10^9^/L, erythrocyte count of 1.94 × 10^9^/L, hemoglobin of 55 g/L, glucose of 1.05 mmol/L, and total protein of 44.01 g/L. A computed tomography (CT) scan revealed a left-sided pyopneumothorax, a defect in the left main bronchus, and signs of pneumonia in the lower sections of the right lung. The bronchoscopy revealed patent trachea and right bronchi (up to segmental) with movable orifices. On the left, the upper lobar bronchus and the lower lobar bronchus were not differentiated. The CT scan showed that the bronchoscope “dropped through” into the pleural cavity. A chest X-ray was not performed.

**Fig. 1:**
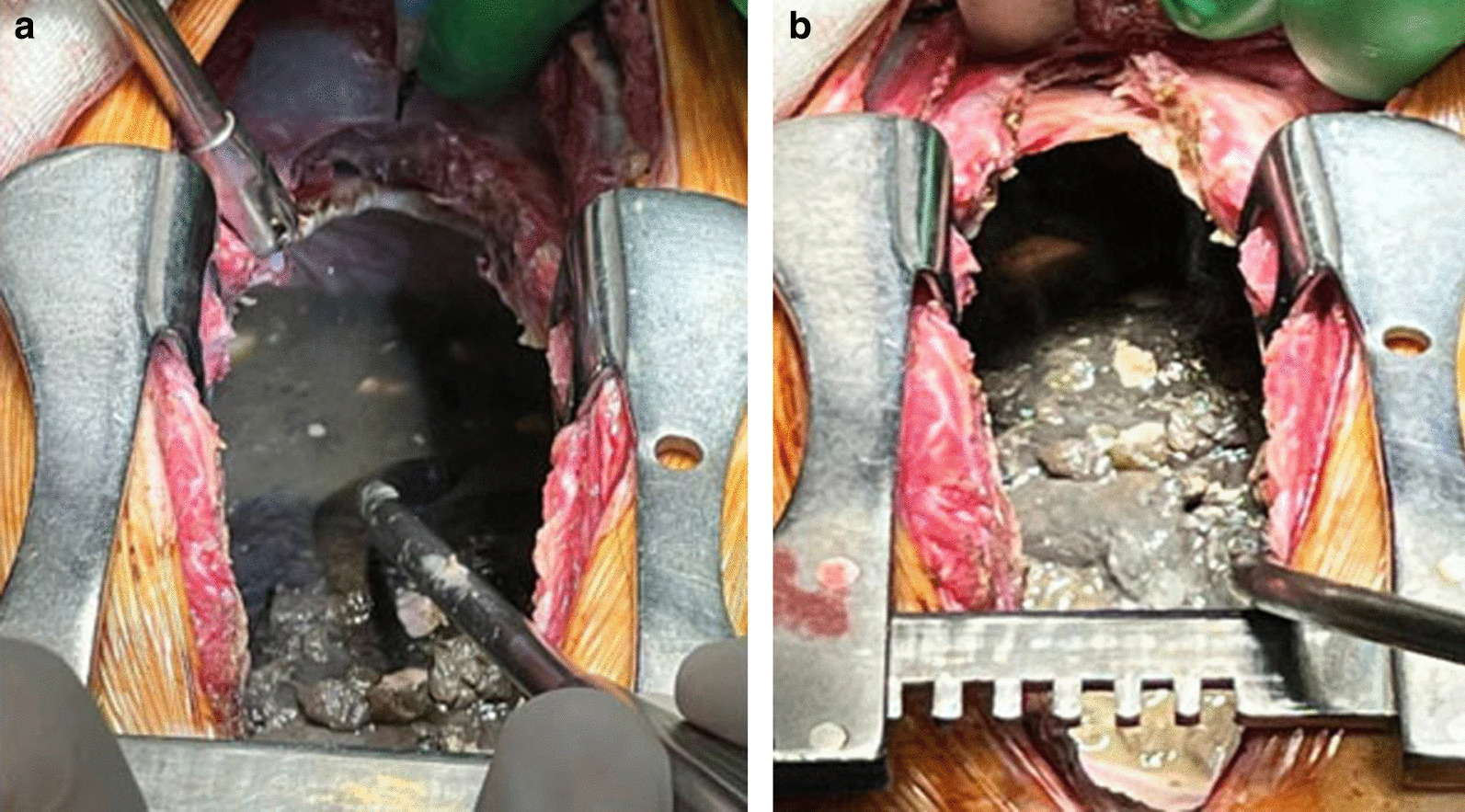
View of the left pleural space (and left lung) after thoracotomy. (**a**) view before the evacuation of the effusion from the pleural cavity; (**b**) view after evacuation of effusion

**Fig. 2:**
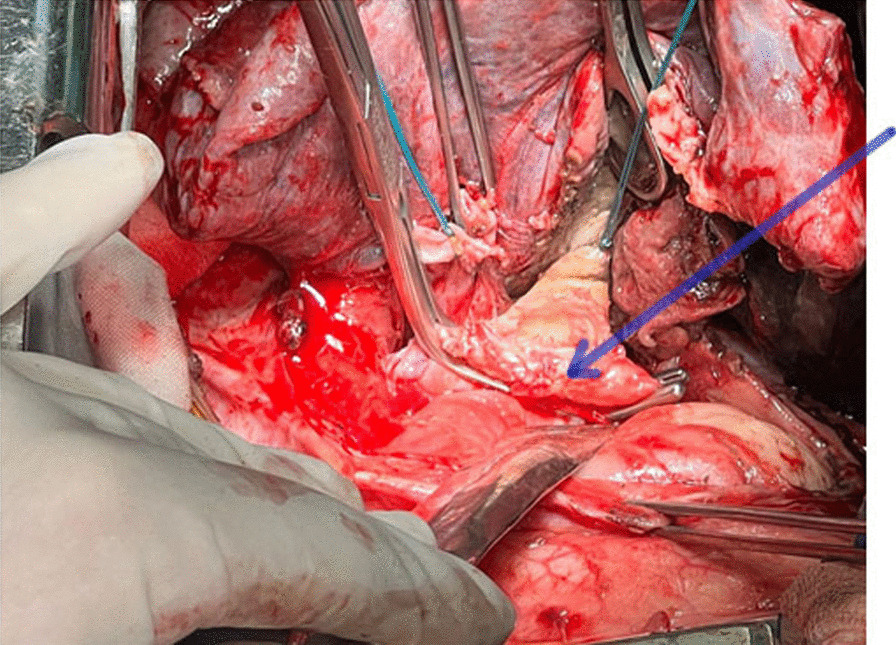
In the background of destruction, self-amputation of the lower pulmonary vein occurred; Satinsky clamp on the right atrium (blue arrow)

Given the anatomy of this area and the proximity of the pulmonary artery to the wall of the necrotic left main bronchus, this situation was regarded as a high risk of arrosive bleeding from the pulmonary artery. The patient was urgently taken to the operating room, and a left thoracotomy was performed. Intraoperatively, gangrene of the left lung with circular necrosis of the left main bronchus (LMB) and diastasis of its edges and pleural empyema on the left were revealed (Fig. 1). We performed pneumolysis, necrectomy, pericardiolysis, and separate intersection of the upper and lower pulmonary veins. It was found that the integrity of the left pulmonary artery (LPA) was maintained. No wall defects were found. The left inferior pulmonary vein (LIPV) was destroyed. The defect in its lumen was obturated with a clot. Bleeding occurred after the removal of a clot from the lumen of the veins (Fig. 2).

**Fig. 3:**
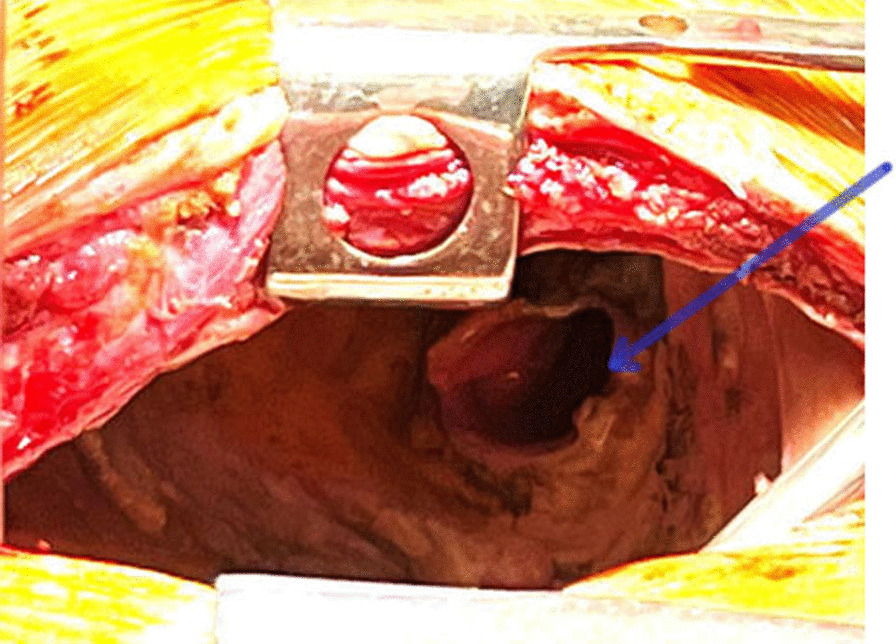
View of the left pleural cavity after removal of the lung and sanitation of the pleural cavity (the blue arrow indicates the stump of the left main bronchus)

**Fig. 4:**
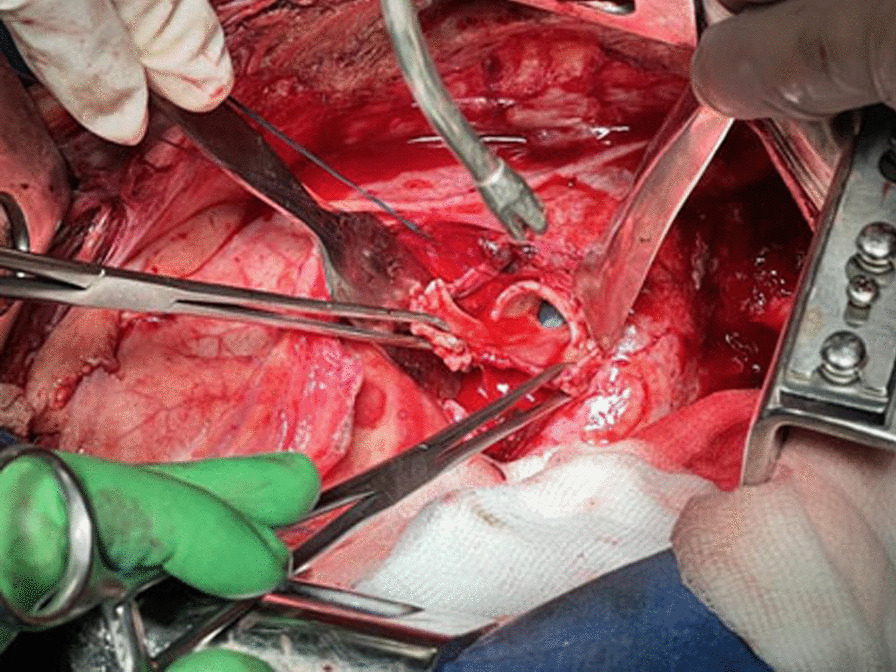
Resection of the self-amputated stump of the left main bronchus, one half of the ring is left

The LIPV was ligated with a vascular stapler; the LPA was crossed and ligated with a vascular stapler. The LMB with a defect was mobilized to the level of the bifurcation and removed further, leaving one half of the ring (Fig. 3). It was repaired with interrupted Biosyn 3/0 sutures without strengthening with autologous tissue (Fig. 4).

**Fig. 5:**
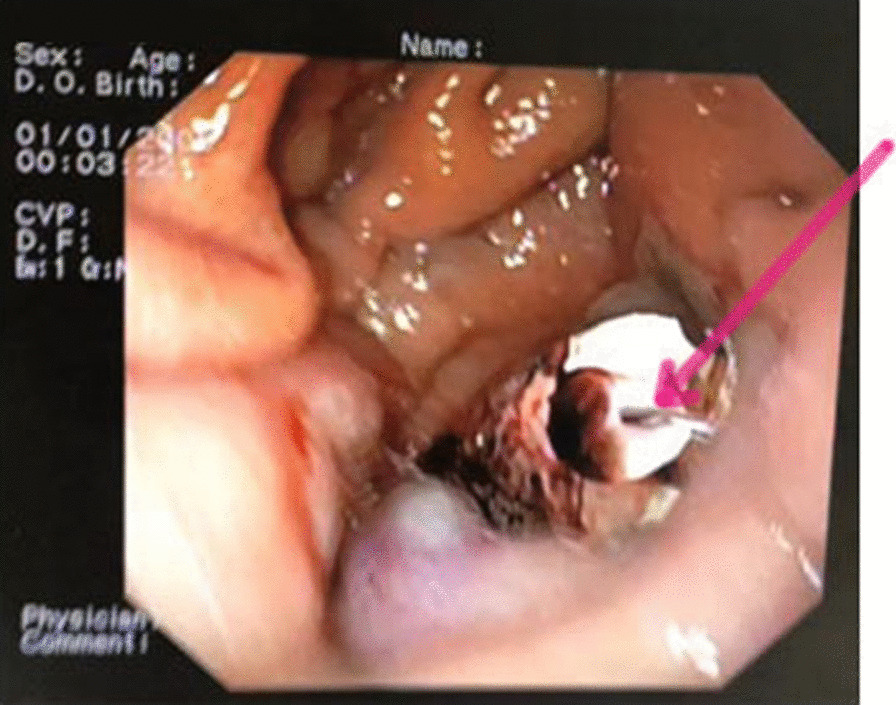
Intraoperative gastroscopy [in the center of the image (red arrow). Instrument located in the left pleural cavity]

**Fig. 6:**
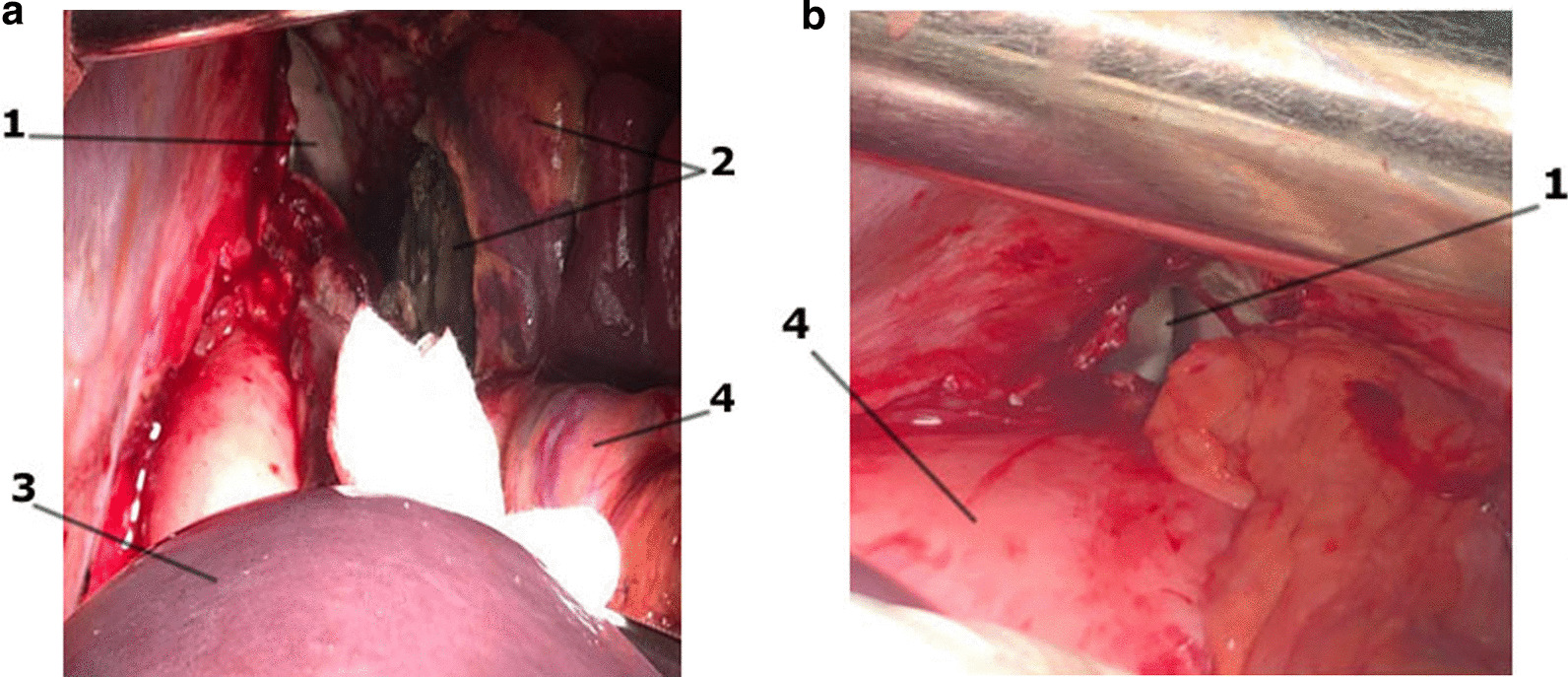
Abdominal stage. **a** View after laparotomy; **b** View after sanitation and splenectomy. 1—Defect in the left dome of the diaphragm; 2—spleen with an abscess cavity; 3—liver; 4—stomach

**Fig. 7:**
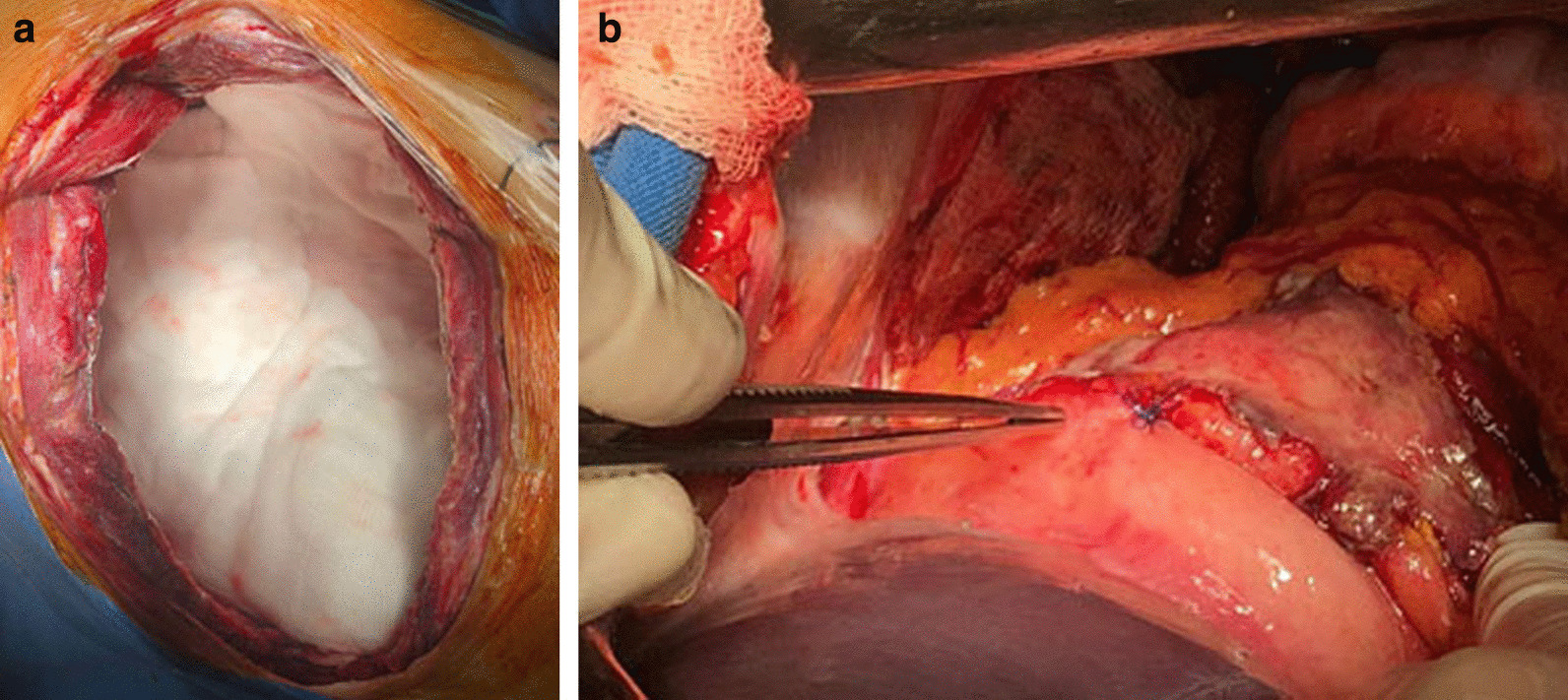
Final view of the operating field. **a** View from the chest wound; **b** View from the side of the abdominal cavity

Also, during the thoracic stage, a defect was found in the left dome of the diaphragm. When performing intraoperative gastroscopy, the optical tip of the apparatus appeared in the surgical field through a defect in the diaphragm (Figs. 5, 6, 7).

The next steps included laparotomy, excision, and suturing of the gastric fundus defect, followed by splenectomy (a “contact” abscess was found in the upper pole of the spleen). The defect of the diaphragm was not sutured; it was covered with Aquacel Ag Foam adhesive; the pleural cavity was tightly filled with gauze tampons. The abdominal cavity was sutured tightly.

Perioperative diagnosis was as follows: perforation of the gastric fundus ulcer with the formation of subphrenic abscess, gastropleural fistula, gangrene of the left lung with self-amputation of the LMB, left-sided pleural empyema.

**Fig. 8:**
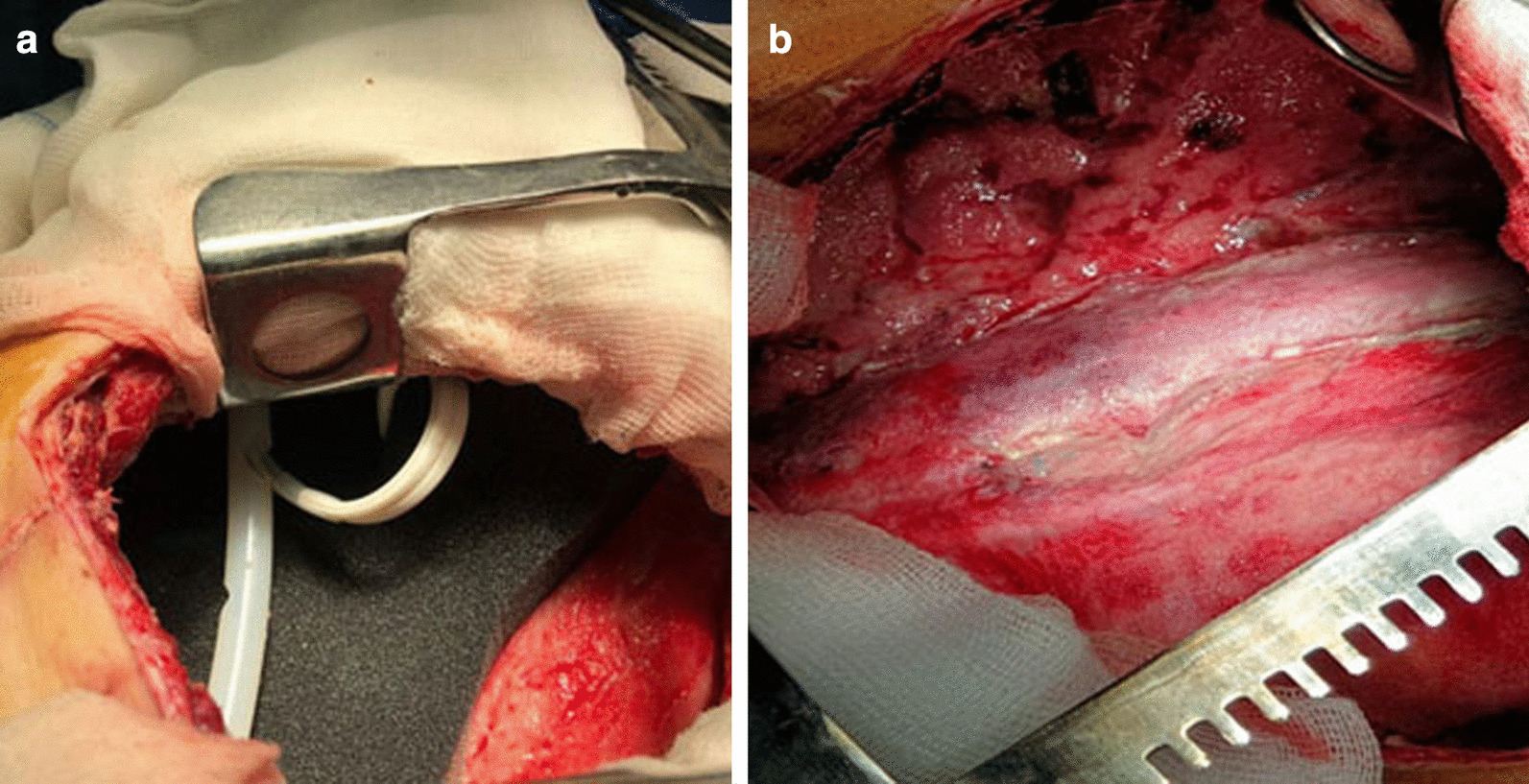
Type of wound: **a** the third dressing; **b** view before suturing (fourth dressing)

During the second stage, we removed gauze tampons from the pleural cavity, and repeated (temporary) the tamponade for 3 days (Fig. 8). During the third stage, we removed all tampons from the pleural cavity and the defect of the diaphragm was sutured.

The patient received prolonged artificial respiration for 1 day; he was extubated in the intensive care unit. On hospital day 20, a follow-up examination revealed type 1 diabetes mellitus (first detected). Histological examination revealed progressing gastric ulcer. A CT examination at 1.5 months after surgery demonstrated postoperative changes. One month after being discharged from the hospital, the patient returned to work.

## Discussion

Gastropleural fistula, due to intrathoracic gastric perforation, is an exceptionally rare diagnosis. The gangrene of the left lung as a complication of a perforated ulcer is an extremely rare occurrence. Indeed, similar descriptions of gastropleural fistulas have not been found in the available literature.

The diagnosis is suspected by the presence of appropriate clinical signs (dyspnea, respiratory failure), according to bronchoscopy and gastroscopy, together with radiological signs of hydropneumothorax and pneumomediastinum. The prognosis is poor. However, surgical management involving a thoracotomy and potentially a laparotomy is the only possible way to save these patients. The prognosis of gastropleural fistulas depends on the etiology of the fistula, early diagnosis, and prompt management [1, 2, 4]. The mortality and morbidity are associated with the extravasation of corrosive gastric contents exuding into the pleural and peritoneal cavities, and the nutritional deficit occurring due to the perforation [1, 5].

In the present case, the decision to operate was made due to the extremely high risk of internal bleeding: the purulent-necrotic cavity around the LMB is in contact with the “aortic window,” which creates a high risk of purulent fusion of the main vessels in this area.

## Conclusion

Although, radical surgery may be preferable for suspected malignancy, it should be weighed carefully against the risk of sepsis and the morbidity associated with a prolonged procedure in a sick patient [6, 7]. If the risk of bleeding and infection has been reduced, damage-control surgery may be an acceptable alternative in severely ill patients, with more extensive resection reserved for later [3].

## Data Availability

Data are available from the corresponding author on reasonable request.

## Abbreviations

LMB: Left main bronchus
LIPV: Left inferior pulmonary vein
LPA: Left pulmonary artery
CT: Computed tomography
